# The Big One: How We Must Prepare for Future Deadly Pandemics

**DOI:** 10.3201/eid3205.252036

**Published:** 2026-05

**Authors:** Courtney N. Dillingham, Gary A. Brooks, Yolanda M. Brooks

**Affiliations:** US Customs and Border Protection, Washington, DC, USA (C.N. Dillingham); Retired, Bradenton, Florida, USA (G.A. Brooks); Pennsylvania Department of Health, Harrisburg, Pennsylvania, USA (Y.M. Brooks)

**Keywords:** outbreaks, communicable diseases, epidemiology, humans, pandemics, public health surveillance, COVID-19, respiratory infections, severe acute respiratory syndrome coronavirus 2, SARS-CoV-2, SARS, coronavirus disease, coronavirus, bacteria, influenza, viruses, zoonoses, bioterrorism and preparedness, book review

*The Big One: How We Must Prepare for Future Deadly Pandemics* is the second book by authors Michael Osterholm and Mark Olshaker discussing politics, reviewing previous pandemic responses, and summarizing infectious disease microbiology to guide pandemic preparedness (Figure). Osterholm writes with authority from his decades of experience in public health and epidemiology at the Minnesota Department of Health and the University of Minnesota Center for Infectious Disease Research and Policy Research and Innovation. As a bestselling author and award-winning filmmaker, Olshaker displays his talent for engaging storytelling. Combining their skillsets, the authors lead readers through 8 chapters of storytelling and discussion across a myriad of interrelated topics covering the evolution of a viral pathogen of pandemic potential, epidemiology of airborne transmission, mandates, medical interventions, effective communication, policy, and next steps.

**Figure:**
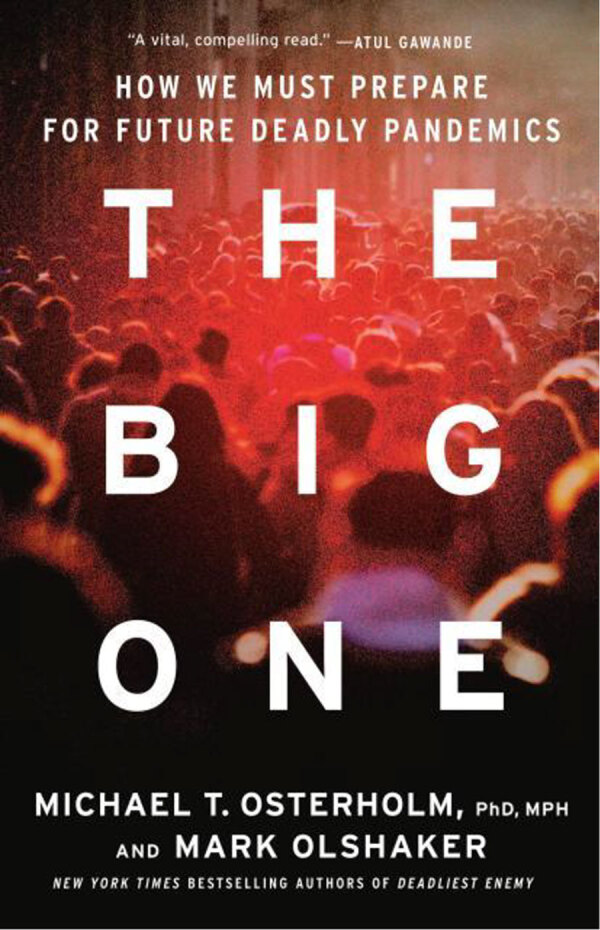
The Big One: How We Must Prepare for Future Deadly Pandemics

Each chapter begins with an excerpt of a fictional yet familiar story that catches the reader’s attention by detailing how a novel respiratory pathogen, starting at patient zero, becomes a global pandemic. The story highlights our interconnectedness by describing how a novel pathogen can travel globally before medical or public health communities are aware of its existence. Focusing on the United States, the authors also discuss essential aspects of pandemic response such as vaccine development, air quality and masks, reliable and actionable public health surveillance, effective communication, mandates, and pandemic-related policy.

Each chapter provides a foundational overview of topics such as the epidemiology of airborne transmission, history of public health surveillance, types of vaccinations and their limitations, evolution of viral pathogens, and diagnostic tests for respiratory viruses. Using an evidence-based approach, the authors explain, recommend, and critique previous pandemic responses, particularly COVID-19. Critiques and recommendations are often accompanied by references to scientific literature, subject matter experts, and historical references. The authors are explicitly critical of actions not supported by rigorously evaluated evidence, such as the Emergency Use Approval by the US Food and Drug Administration of the BinaxNOW COVID-19 point-of-use lateral flow diagnostic tests (Abbott, https://www.abbott.com), which were approved on the basis of just 117 positive samples. The authors also praise the rapid and accurate clinical and laboratory surveillance metrics for COVID-19 in countries with centralized healthcare, such as the United Kingdom, Israel, and Canada. The goal of this analysis is to glean lessons from planning failures and mistakes made in previous pandemic responses, as well as to provide a blueprint for mitigating damage from the next pandemic. The authors end each chapter with concise and direct takeaways.

The authors have some self-biases; Osterholm recalls instances where he correctly provided warnings and recommendations that others dismissed. The positive contributions of the Center for Infectious Disease Research and Policy Research and Innovation are also discussed, but little if any criticism is included.

The book is a worthwhile read for professionals in public health, healthcare administration, life sciences, public policy, emergency preparedness, and anyone interested in pandemic preparedness. It provides readers with an opportunity to reflect critically on the interdisciplinary facets of pandemic preparedness and response. The book could serve as a reference guide throughout one’s career to reflect on how to incorporate evidence-based strategies to respond to major societal concerns.

